# Long-term drift behavior in metal oxide gas sensor arrays: a one-year dataset from an electronic nose

**DOI:** 10.1038/s41597-025-05993-8

**Published:** 2025-10-08

**Authors:** Julius Wörner, Jonas Eimler, Miriam Pein-Hackelbusch

**Affiliations:** Institute for Life Science Technologies (ILT.NRW), OWL University of Applied Sciences and Arts, 32657 Lemgo, Germany

**Keywords:** Scientific data, Chemistry

## Abstract

Although electronic nose technology has been studied for years, drift effects remain one of the major challenges. While ongoing research focuses on effective correction methods, the evaluation of these methods requires reliable and well-documented datasets. However, only a few drift datasets are available, some of which lack sufficient experimental detail or are outdated. This motivated us to introduce a new long-term drift dataset. It has been collected over 12 months using a commercial electronic nose, which is based on 62-metal oxide sensors. The measurements were conducted under controlled experimental conditions with three analytes (diacetyl, 2-phenylethanol, and ethanol) in different concentrations. The dataset consists of 700 time-series recordings, for which we provide both the raw data and a set of pre-extracted features. The data can support the development, evaluation, and comparison of methods for feature extraction and selection, as well as drift detection and compensation. By providing a comprehensive, well-documented dataset, we aim to advance research on sensor drift in electronic nose systems.

## Background & Summary

Over the last decades, electronic nose technology (E-noses) has advanced significantly especially through enhanced sensor materials and machine learning algorithms^1,2^. Hence these multi-sensor systems have been explored in many areas, including agriculture, healthcare, security, the food industry, and water sectors^2^.

E-noses aim to mimic the human olfactory system to sense and detect gaseous mixtures^2,3^. Therefore, the application of both cross-sensitive sensors, covering a broad spectrum of volatile substances, and machine learning methods is required^4^. To develop and train appropriate pattern recognition systems, it is essential to extract characteristic and robust information from the sensor responses^3^. However, responses from metal oxide gas sensors are especially susceptible to both short- and long-term drift effects^5–7^. Physical and chemical alteration of the sensor material lead to the unpredictable gradual change of the gas sensor’s response, regardless of whether the analytical conditions or the analyt’s composition are modified^6,8^. Such gradual aging and poisoning of the sensor material is known as first-order drift, whereas uncontrollable variations in experimental conditions, like changes in temperature or humidity, lead to so-called second-order drift effects^8,9^. The sensor drift problem often leads to poor repeatability and reproducibility of E-nose readings^5^. Countermeasures for drift correction are an area of continued research interest^10,11^.

Reliable data sets are crucial for the development and design of drift correction algorithms^6^. Two comprehensive sensor array datasets were released by Vergara *et al*.^8,12^. One of these was collected over 36 months. It consists of 13,910 time-series recordings of 16 metal oxide gas sensors with six different gas analytes. With prolonged time sensor drift and lower performance of the classifiers were observed^8^. Unfortunately, this dataset only provides some pre-extracted features but no raw data, which reduces flexibility for researchers to test their own feature extraction and selection methods^13,14^. The other dataset by Vergara *et al*., published in 2013, consists of 18,000 recordings obtained with a 72-sensor array in a wind-tunnel setup over 16 months. The authors employed an array of nine distinct sensor boards, each containing eight sensors, to perform measurements with ten different analytes. They tested several scenarios with varying wind speeds, operating voltages, and sensor board positions within the wind tunnel^12^. In this setup, differences in proximity to the gas source and the inhomogeneous dispersion of sample air led to unequal gas exposure across sensors. This, in turn, procuded a spatial distribution of long-term sensor baseline drift^6^. The experimental conditions differ markedly from those used in the present study. Then years later, in 2023, Kumar *et al*. published a dataset comprising the recordings of a six-sensor array exposed to 11 different analytes over a 12-month period. However, the accompanying publication provides limited details on the experimental setup, controls, and data access^15^. Only a few other comparable datasets are publicly available, most of which either do not capture long-term drift effects or lack documentation of data acquisition period^16–19^.

Summarized, the review of existing drift datasets reveals two major limitations. First, most datasets are over ten years old and do not reflect the substantial advances in sensor technology over the past decade. Second, many datasets are insufficiently documented, particularyl regarding the experimental design, which hinders their reuse for scientific purposes.

To address these gaps, we generated a long-term dataset comprising measurements from an E-nose equipped with an array of 62 gas sensors. The extensive sensor array offers two main advantages: it increases the likelihood of detecting any given gas, enhancing the system versatility, and it provides more data to construct a more comprehensive and robust ‘fingerprint’ for each analyte^20–22^. Furthermore, we provide detailed information on the experimental setup and data collection process to ensure data reliability and enabling traceable and reproducible data analysis. Specifically, the dataset consists of 700 time-series recordings obtained with a 62-sensor metal-oxide array over a period of 12 months. We focused on three chemical analytes: diacetyl, 2-phenylethanol, and ethanol. This compound selection ensures the presence of different chemical moieties, namely carbonyl functions (diacetyl), hydroxyl groups (ethanol, 2-phenylethanol) and an aromatic site (2-phenylethanol). They also differ physically in their vapor pressures (e.g. pure diacetyl ~ 5,890 Pa at 20 °C; pure 2-phenylethanol ~ 8 Pa at 20 °C)^23,24^. Diacetyl and 2-phenylethanol were measured at two different concentrations with ethanol (as 5% v/v aqueous solution) as diluent. The 5% v/v aqueous solution was also analyzed as sample and used as blank.

The aim of this dataset is to expand the publicly available resources for studying sensor drift in multi-sensor arrays based on metal-oxide gas sensors. It enables the systematical evaluation and improvement of methods for feature extraction, feature selection, aas well as drift detection or compensation. Moreover, the high dimensionality combined with the relatively small sample size offers an opportunity to explore feature selection techniques aimed at identifying subsets that are resilient to long-term drift. By providing both the the raw sensor data and a set of pre-computed features, other, researchers have the flexibility to either extract their own features customized tailored to their approaches or use the given provided features directly to facilitate the development of robust classification algorithms. Finally, varying analyte concentrations enable investigations of detection limit drift in metal-oxide E-nose systems.

## Methods

### Overview of experimental design

The experiments to capture the drift behavior of metal-oxide gas sensors were conducted in a commercially available E-nose system (Smelldect GmbH, Deckenpfronn, Germany). Over a period of 12 months, the analytes were measured. In sum, 117 measurements were performed for each concentration of 2-phenylethanol and diacetyl, and 232 measurements for ethanol samples. The measurements were carried out under laboratory conditions. However, to simulate realistic operating conditions, the system was periodically subjected to additional measurements not included in the present dataset.

### Electronic nose system

**Fig. 1:**
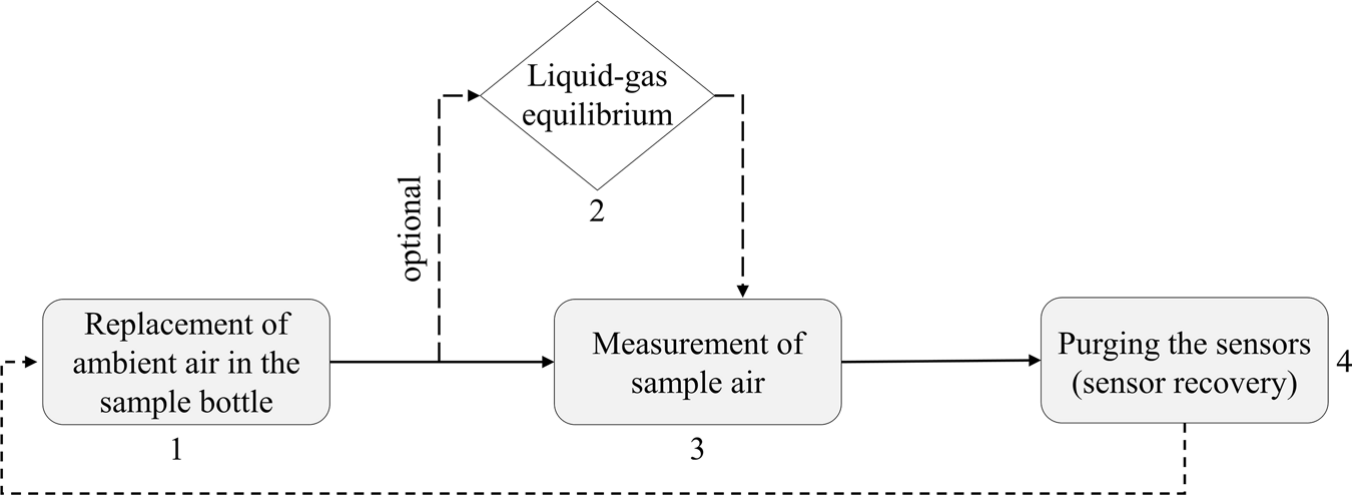
Complete measurement cycle divided into 4 phases. At the beginning, the ambient air in the sample bottle is replaced by compressed air (1), in order to remove volatiles which are not emitted from the sample itself. Subsequently, an optional phase for the establishment of the gas-liquid equilibrium can be implemented (2). After sample measurement (3), the sensors are purged (4) to regenerate the sensor surfaces.

The E-nose used in this study was manufactured by the company Smelldect (Smelldect GmbH, Deckenpfronn, Germany) and features 62 SnO_2_ nanowires, which are excited using ultraviolet light. Additionally, temperature and humidity sensors are included in the array. Instead of ambient air, this system works with compressed air from a gas cylinder. The E-nose system operates in three distinct phases to enable customized measurement protocols. Gas flow routing differs between phases to facilitate sensor purging, sample analysis, and correct baseline measurements. Both phases 1 and 4 typically serve as baseline recording periods, phase 3 consists of the sample measurement. Phases 1 and 4 direct the carrier gas exclusively to the sensor chamber, achieving the regeneration of the sensor surface through purging and the establishment of a liquid-gas equilibrium in the sample bottle. Phase 2 additionally flushes the sample bottle, replacing ambient air. Phase 3 introduces sample air into the sensor chamber for analyte detection. Figure 1 illustrates a typical measurement workflow.

Purging the sample bottle with compressed air before the start of a new cycle reduces the influence of other volatiles, which are present in ambient air. The baseline readings are recorded during purging, as the clean compressed air is routed over the sensors. Baseline measurements can be conducted simultaneously with gas-liquid equilibrium establishment, as only purified compressed air is in contact with the sensor. To control the E-nose measurements, the software supplied by Smelldect (Kamina Observer version 2.0, Karlsruher Institute for Technology (KIT), Karlsruhe, Germany, 2013-202) was used.

### Preparation of the sample concentrations

In this study, three different substances were used: diacetyl (Butane-2,3-dione; 99%, Thermo Scientific Chemicals, Waltham, MA, USA, Cat. No.: A14217.22, Lot: 10246588), 2-phenylethanol (98+ %, Thermo Scientific Chemicals, Waltham, MA, USA, Cat. No.: A15241.30, Lot: 10233538), and ethanol (99.5% denatured with 1% butanone, VWR Chemicals, Radnor, PA, USA, Cat. No.: 85033.460, Lot: 22A104031). Throughout the experiment, 2-phenylethanol and diacetyl were stored at 5 °C in the refrigerator. The dilution series were freshly prepared each day before measurement using an LLG Labware pipette (100–1000 μL single channel pipette, Lab Logistics Group GmbH, Meckenheim, Germany). Ethanol was diluted in deionized water to a concentration of 5% v/v. Diacetyl and 2-phenylethanol were diluted in the ethanol (5% v/v) solution. Concentrations of diacetyl and 2-phenylethanol were selected to provide one level substantially above the limit of detection (LOD) and another marginally above the LOD for each analyte. The LODs were obtained in ealier investigations^25^. The aqueous ethanolic solution (5% v/v) served as sample and as blank. All indicated concentrations for every substance refer to the concentration in the solution.

### Experimental setup and data collection methodology

**Fig. 2:**
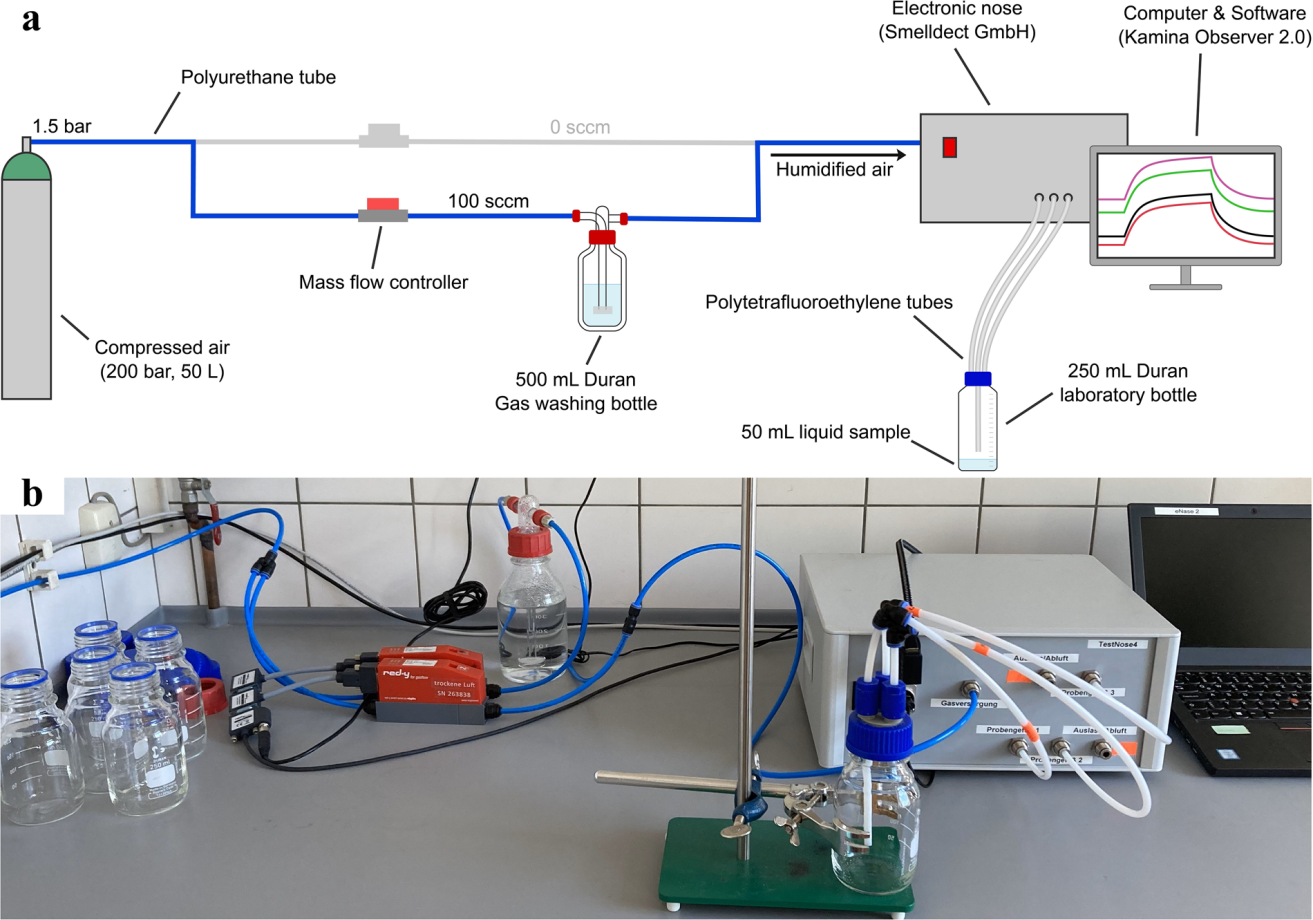
Process flow chart (**a**) and photo (**b**) of the experimental setup. From left to right: the connection to the compressed air cylinder for carrier gas supply, mass flow controllers to regulate gas flow rates and adjusting the relative humidity of the carrier gas, a gas washing bottle for humidifying the gas stream, a 250 mL sample bottle, and the electronic nose system for measuring the volatile compounds. The system is built as a closed system and allows measurements to be taken separated from the ambient air.

To minimize inter-day variability, sensors underwent 24-hour pre-conditioning with dry compressed air without intermediate measurements. At the beginning of each day, the system was connected to an empty sample bottle for approximately 90 min until a stable temperature in the sensor chamber was reached. This minimized the influence of initial temperature drift on the first measurements. On each day, the measurements were carried out in the exact same order of analytes and concentrations thereof as triplicates to minimize influences other than time-related long-term drift effects. All 18 sample measurements of a day were performed consecutively. The measurement cycle, and thus the duration of a single sample measurement, was set to 20 minutes. This time includes sensor purging. The order of the measurements was as follows: 3x ethanol 5% v/v, 3x 0.1 ppm diacetyl, 3x 1 ppm diacetyl, 3x ethanol 5% v/v, 200 ppm 2-phenylethanol, 3x 1000 ppm 2-phenylethanol. Compressed air (Air, compressed, UN1002, Westfalen AG, Muenster, Germany) was used as a carrier gas at a flow rate of 100 sccm (mass flow controllers: Smart Controller GSC, red-y smart series, Voegtlin Instruments GmbH, Muttenz, Switzerland). The carrier gas was humidified using a gas washing bottle filled with deionized water. The humidity in the sensor chamber was set between 70 to 80%, depending on the exact temperature of the system. The sample bottle had a total volume of 250 mL. The sample bottles were filled with 50 mL of each dilution prior to measurement. Within the triplicate measurement design, the first volume of liquid was measured twice consecutively before being replaced for the third measurement. In this way, repeatability and reproducibility were included, capturing intra-sample as well as inter-sample variability. Potential carryover effects between consecutive measurements were thus reflected. The tubes and lids were not replaced during the entire one-year period. The experimental setup is shown in Fig. 2.

### Computational processing and feature extraction

The E-nose system software generates a .csv file containing the daily collected resistance data of all samples. These times-series were separated to obtain one file per measurement with comprises of all phases including resistance values for all sensors. Failed, technically incorrect or protocol-deviant measurements were excluded from analysis.

## Data Records

**Fig. 3:**
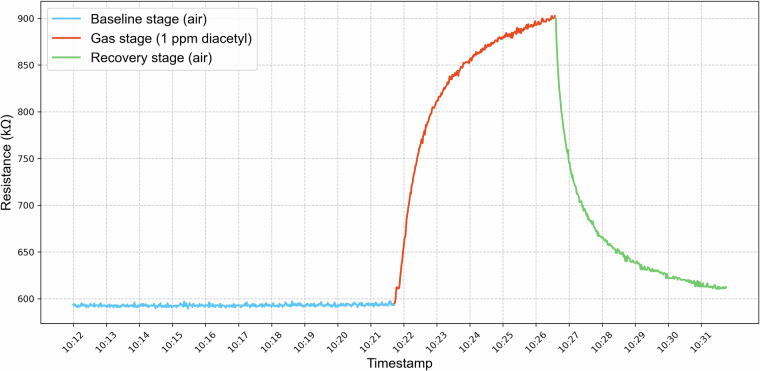
A complete measurement cycle in which the resistance of sensor 2 is recorded for diacetyl at a concentration of 1 ppm. Resistance measurements were acquired on the initial day of data collection (2024-02-16). The colors indicate the individual stages.

The dataset can be found at Zenodo, 10.5281/zenodo.15681119^26^. All data files are available in .csv format with a single space character as separator for the values within a row and a period as decimal separator. Each file contains the measured values for one sample. The first row of each file contains the headings of the columns, with the following columns: timestamp, date, sensor resistance readings from all sensors (for gas, humidity, and temperature sensors), and corresponding measurement cycle stage (1, 2 or 3, as shown in Fig. 3). The gas sensors’ resistance values are marked with a preceding ‘R’, the temperature sensor with a ‘T’ and the humidity sensor with a preceding ‘H’. For example the resistance of the sensor number 1 is marked as ‘R1’. Table 1 illustrates the structure of the raw data .csv files, using the example of the first diacetyl measurement at a concentration of 0.1 ppm from day 1.

**Table 1:** An overview of the file structure present in the .csv output files.

Time[hh:mm:ss]	Date[yyyy-mm-dd]	R1[Ohm]	…	R62[Ohm]	T01[degC]	H01[%rh]	Cycle_Stage
09:12:00	24-02-16	591887	…	4377056	39.65	71.2	1
…	…	…	…	…	…	…	…
09:31:58	24-02-16	596264	…	4411631	39.85	71.3	3

Every file contains columns for the timestamp, date, resistance sensors with preceding ‘R’, temperature sensor with preceding ‘T’ and humidity sensor with preceding ‘H’, as well as the cycle stage in which the measurement was performed.

For each sensor signal, the response curve can be divided into three stages (Fig. 3). The first one represents the baseline stage, where only clean compressed air is measured (1). The second one is the gas stage, in which the sensor is exposed to the sample air (2). During the following recovery stage, the sensor array is again purged with clean compressed air (3). In our dataset, the cycle stage for each data point is stored in a separate column (see exemplarily Table 1).

**Fig. 4:**
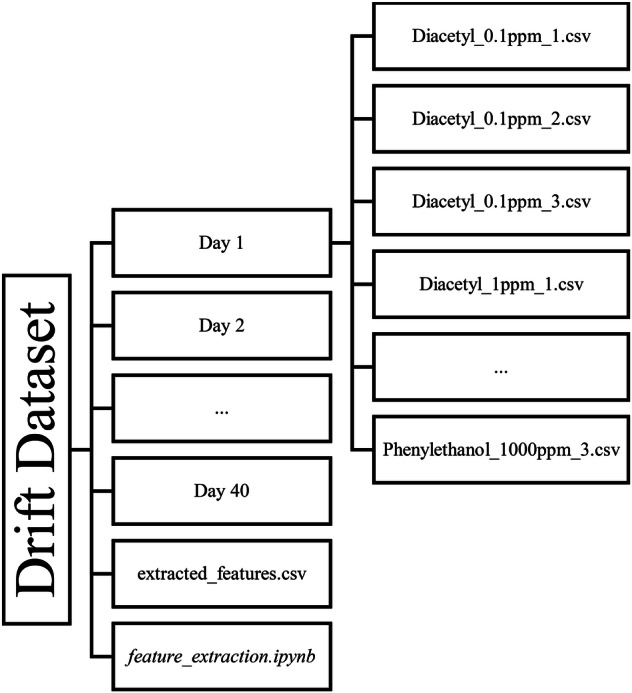
An overview of the folder structure and naming conventions in the dataset. The dataset contains 39 daily subfolders with raw measurement data, a jupyter notebook and an *extracted_features.csv* file containing all extracted features. The daily folder stores individual measurements as .csv files named by substance and concentration (e.g., *Diacetyl_1ppm_1.csv* for the first measurement of the triplicate of 1 ppm diacetyl).

The folder structure is as follows: The main folder contains 39 subfolders (as day 14 was discarded due to deviations from the protocol), each of which contains all the raw data from the individual measurement days. The .csv file called *extracted_features.csv* contains the processed data with the extracted features listed in Table 2. Each folder consists of the raw data of all 18 measurements of the respective day as single .csv files. The files are named according to the measured substance and its concentration. For example, the file named *Diacetyl_1ppm_1.csv* corresponds to the first measurement (of the triplicate) of the substance diacetyl with a concentration of 1 ppm. For the 5% v/v ethanol solution (named as EtOH), the measurements are numbered in ascending order within the whole day. All files are consistently labeled according to the described naming convention, which encodes the class information directly in the filename. Consequently, the label itself is not redundantly stored within each raw data file. In the *extracted_features.csv* file, the class label for each individual measurement is stored in the column named *Class*. The sample classes (defined as the combiantion of substance and concentration) are always explicitly annotated, ensuring that the dataset is suitable for supervised classification tasks. In total, the dataset contains 700 measurements. Figure 4 sums up the folder structure and file naming conventions.

**Table 2:** Overview of the features extracted from the time series of all sensors.

Name of the Feature	Formula	Description
Mean Resistance	${\bar{R}}_{{sample}}$	Mean value calculated from the last 10 resistance values during the sample measurement stage (i.e., stage 2), representing the final sensor response when exposed to sample air.
Relative Difference	$\frac{({\bar{R}}_{{sample}}-{\bar{R}}_{{baseline}})}{{\bar{R}}_{{baseline}}}$	Mean values calculated from the last 10 values of the respective stage. The feature quantifies the sensor’s response to sample air relative to its baseline level.
Recovery Level	$\frac{{\bar{R}}_{{baseline},{pre}}}{{\bar{R}}_{{baseline},{post}}}$	Recovery level defined as the ratio between the baseline mean before sample exposure and the baseline mean after sample exposure. It indicates the sensor’s ability to return to its original baseline, serving as a measure of recovery and signal drift.
Sample Start-Slope	$\frac{\mathop{\sum }\limits_{i=1}^{30}\left({t}_{i}-\bar{t}\right)\ast ({R}_{i}-\bar{R})}{\mathop{\sum }\limits_{i=1}^{30}{\left({t}_{i}-\bar{t}\right)}^{2}}$	The sample start-slope defined as the slope of the linear regression for the first 30 values during sample air exposure, reflecting how fast the sensor responds to the sample in the first seconds. We used t = i.

R represents the resistance of each sensor in Ω (Ohm). The sub-index t denotes the index of the measurement points, starting from the first recorded value of each measurement. Resistance values are grouped as follows: baseline (clean air before sample air exposure), sample (during sample air exposure), and pre/post (clean air immediately before and after sample air exposure, respectively).

## Technical Validation

The experimental design ensures the reduction of random influences to a minimum. Commercially available, compressed air from a pressurized cylinder provided consistent carrier gas quality, eliminating ambient air contamination. Defined humidity control provided standardized carrier gas conditioning in contrast to humidity fluctuation of ambient air.

The datasets of the sensor array were inspected for missing data recordings. The plausibility and recording of the temperature and humidity readings were checked repeatedly. Incorrect sample measurements were removed from the dataset. Due to incorrect measurements, the 14^th^ measurement day was eliminated from the data set. The label of the sample measurements was entered into the software immediately after completion of the respective measurement. We calculated the signal-to-noise ratio (SNR) for all sensor measurements using the following formula:

1 $${SNR}=\frac{\left|{\bar{R}}_{{sample}}-{\bar{R}}_{{baseline}}\right|}{{{\rm{\sigma }}}_{{baseline}}}$$

where ${\bar{R}}_{{baseline}}$ and ${\bar{R}}_{{sample}}$ are the mean values of the baseline and sample air readings, and ${{\rm{\sigma }}}_{{baseline}}$ is the standard deviation of the baseline. The formula is based on the definition of the detection limit using the SNR described by the International Council for Harmonisation^27^, where the factor 3 is generally accepted for estimating the minimum concentration that can be reliably detected. Based on this definition, the value indicates whether the response of a single sensor can be considered a reliable detection of sample air. Low SNR values does not necessarily imply reduced reliability of sensor readings. However, if a sensor shows low values across all classes, the sensor might be less relevant for substance detection. Conversely, high SNR values suggest that the sensors are indeed responding to volatile compounds emitted from the sample. Across all measurement days, we confirmed that each sensor achieved SNR values greater than 3 for at least one class, demonstrating that every sensor responds to at least one substance. More than 81% of all sensor measurements across all classes reached SNR values ≥ 10, indicating the presence of strong signals.

Sensor chamber conditions were continuously monitored throughout the measurements using integrated temperature and relative humidity sensors. The temperature range was between 37.95 °C and 43.15 °C, with a mean of 40.38 °C and a standard deviation of 1.17 °C. This indicates that there were no unusual or extreme temperatur readings during the experiments. The maximum coefficient of variation for the temperature within a single measurement cycle was 0.32%, confirming stable temperature conditions during the individual measurements. The values for relative humidity ranged from 69.50% to 81.70% with a mean of 76.00% and a standard deviation of 3.44%. This shows that defined conditions were recorded over the 12-month measurement period.

To quantify baseline reproducibility, short- and long-term variations were investigated using the coefficient of variation (CV) of the baseline for each sensor^6^. The CV is defined as the ratio of the standard deviation σ to the mean μ. For each sensor, the baseline was determined by first calculating the mean of the last 20 resistance values of cycle stage 1 (Fig. 3), prior to sample gas release. The overall mean and standard deviation was then computed across these baseline means, either for individual days or for the entire period. The analysis revealed long-term CVs ranging von 25.74% to 40.77%, depending on the sensor. On the other hand, the short-term CV was considerably lower, between 0.38% and 1.21%. These baseline variabilities for all sensors over time indicate the presence of drift effects. The correlation between the long- and short-term CVs was weak (r = −0.08).

To ensure the same initial conditions for the sensors each day, the system was purged with clean compressed air the day before the real measurement and thus the sensors were preconditioned. We therefore assume that the sensors did not directly react to previously measured samples, but only to the desired substances.

**Fig. 5:**
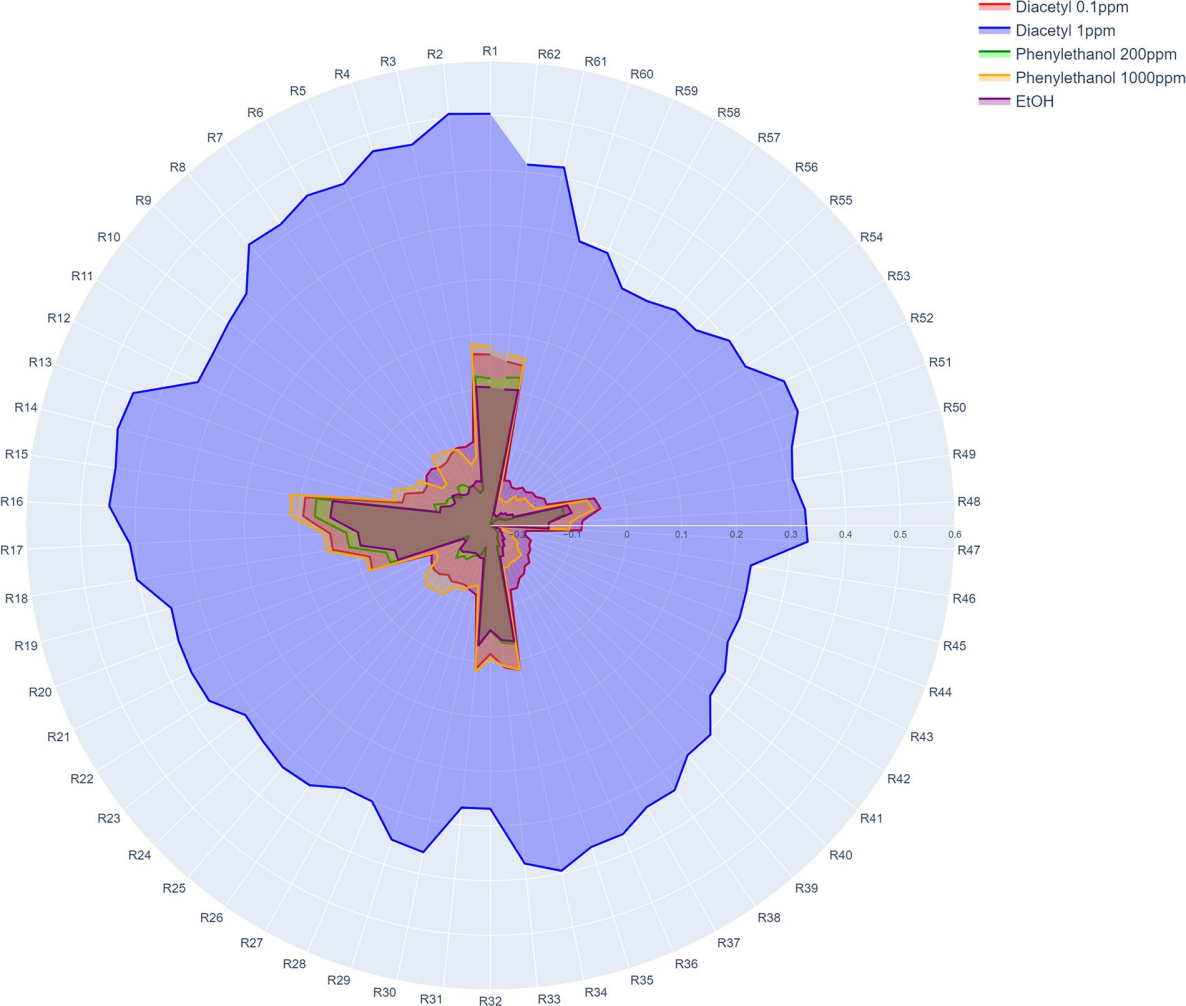
Radar chart illustrating all five classes based on the mean relative difference of all gas sensors (R1-R62) on the first day of measurement. Each axis represents a single sensor, and the plotted areas reflect the sensor response patterns for each class. The distinct areas make clear that the response patterns differ between classes.

To check whether the data set is suitable for classification in general, we created a radar chart (Fig. 5). It shows the volatile signatures depending on the different substances and concentrations.

A potential limitation of the dataset is its restriction to only three main analytes. Thus, despite selecting analytes with diverse chemical moieties and possibly drift inducing abilities, our investigation particularly excludes complex gas mixtures, indicating the need for broader drift analysis across additional chemical classes. However, our selection was guided by both, practical and methodological considerations. At first, the used analytes demonstrate broad applicability in food aroma profiling, fermentation monitoring, brewing processes, and quality assessment^28–31^. Second, the selected analytes cover different response characteristics and the further inclusion of two concentrations of both substances allows the distinguishability of low concentrations. Third, prior studies characterized sensor responses and detection limits for these compounds, confirming that all sensors show analyte-specific responses, facilitating reliable detection and data comparison^25^.

In sum, the dataset provides valuable opportunities to investigate drift behavior in sensor arrays. Further, existing algorithms can be improved, balancing experimental feasibility with measingful algorithmic evaluation. This dataset can serve as a useful complement to both, newly generated and existing datasets.

## Data Availability

The dataset generated during the study is openly available in the Zenodo repository with the identifier 10.5281/zenodo.15681119 (10.5281/zenodo.15681119).

## Code availability

The code for loading the measurments and extracting the calculated features is provided as a Jupyter Notebook (Jupyter Notebook v6.4.8, Python 3.9.12) named *feature_extraction.ipynb* alongside the dataset. The notebook, tested on Windows 11, contains all necessary functions to import the data from the supplied *.csv* files, compute the features, and export them as a space-separated .*csv* file. Users can select which features to extract and extend the workflow with custom features. The implementation relies on *pandas* (v 1.4.2), *scipy* (v1.7.3), as well as standard modules (i.e., *os, inspect, re and pathlib)*. The function *load_data_from_main_folder* imports all files and stores them as a list of data frames. The *FeatureExtractor* class can then be initialized with the desired features, as well as the input and output directories. This class contains all functions required for feature computation. The method *Extract_features_from_multiple* allows then to calculate all selected features and aggregate them into a single data frame, which can subsequently be exported.
